# Correction: Single Nucleotide Polymorphisms Reveal Genetic Structuring of the Carpathian Newt and Provide Evidence of Interspecific Gene Flow in the Nuclear Genome

**DOI:** 10.1371/journal.pone.0101352

**Published:** 2014-06-26

**Authors:** The PLOS ONE Staff

## Notice of Republication

This article was republished on June 2, 2014, to correct a typographical error in the title. The publisher apologizes for the error. Please download this article again to view the correct version. The originally published, uncorrected article and the republished, corrected article are provided here for reference.

## Supporting Information

File S1 Originally published, uncorrected article.
(PDF)

File S2 Republished, corrected article.
(PDF)
