# Correction: Correction: Single Nucleotide Polymorphisms Reveal Genetic Structuring of the Carpathian Newt and Provide Evidence of Interspecific Gene Flow in the Nuclear Genome

**DOI:** 10.1371/journal.pone.0105029

**Published:** 2014-08-04

**Authors:** The PLOS ONE Staff

File S2 is a duplicate of File S1, the originally published, uncorrected article. The publisher apologizes for the error. Please view the correct File S2 – the republished, corrected article – below.

## Supporting Information

File S2 Republished, corrected article.
(PDF)
