# Supplementary figures and images for: Single Nucleotide Polymorphisms Reveal Genetic Structuring of the Carpathian Newt and Provide Evidence of Interspecific Gene Flow in the Nuclear Genome
Source: PLoS One. 2014 May 12;9(5):e97431. doi: 10.1371/journal.pone.0097431 (PMC4018350; doi:10.1371/journal.pone.0097431)

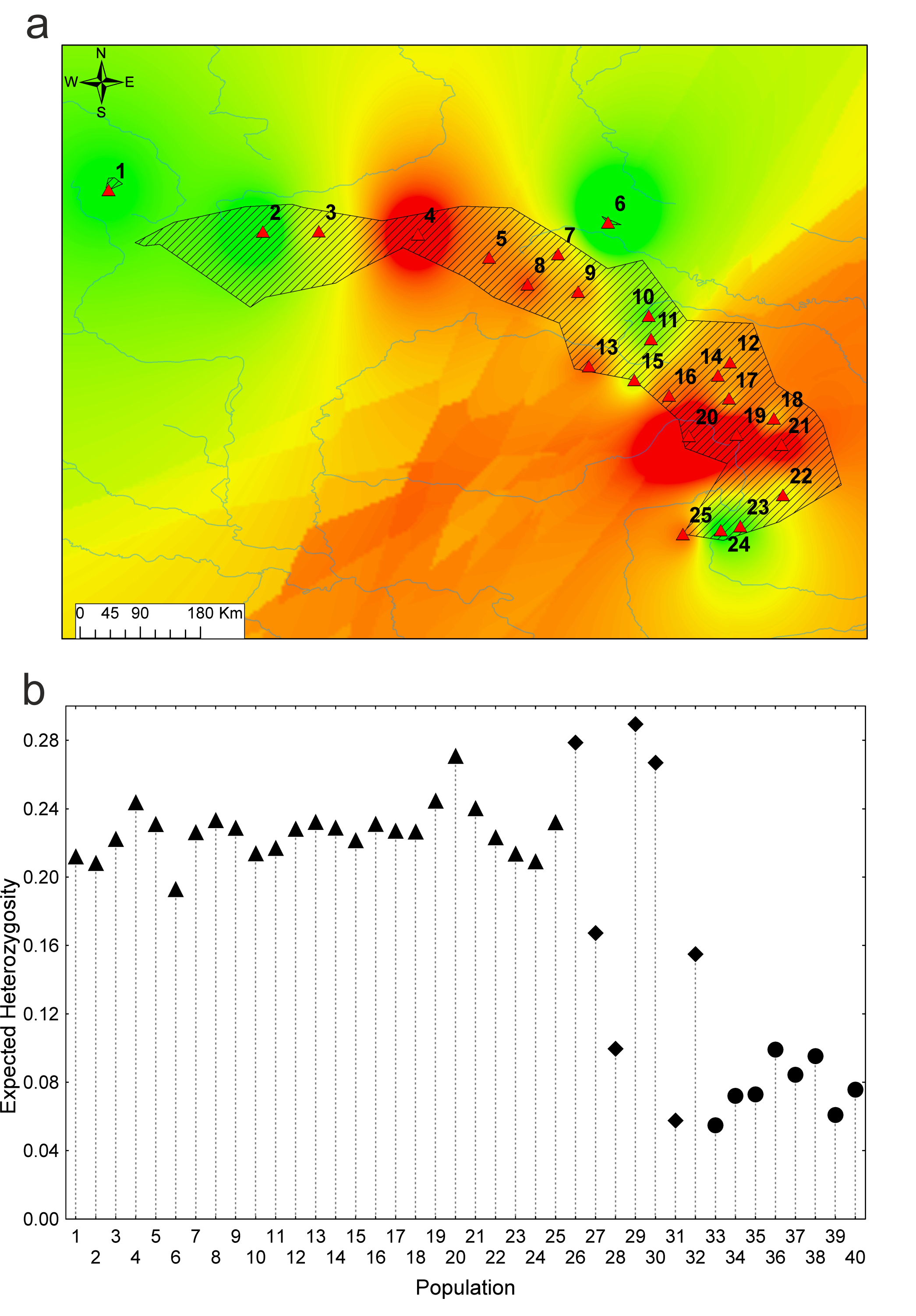

Supplement: Figure S1 — Expected heterozygosity. (a) interpolated geographic gradients in L. montandoni (Lm), (b) means for all populations: triangles – Lm, diamonds – syntopic, circles – Lv. (TIF) [file pone.0097431.s001.tif]

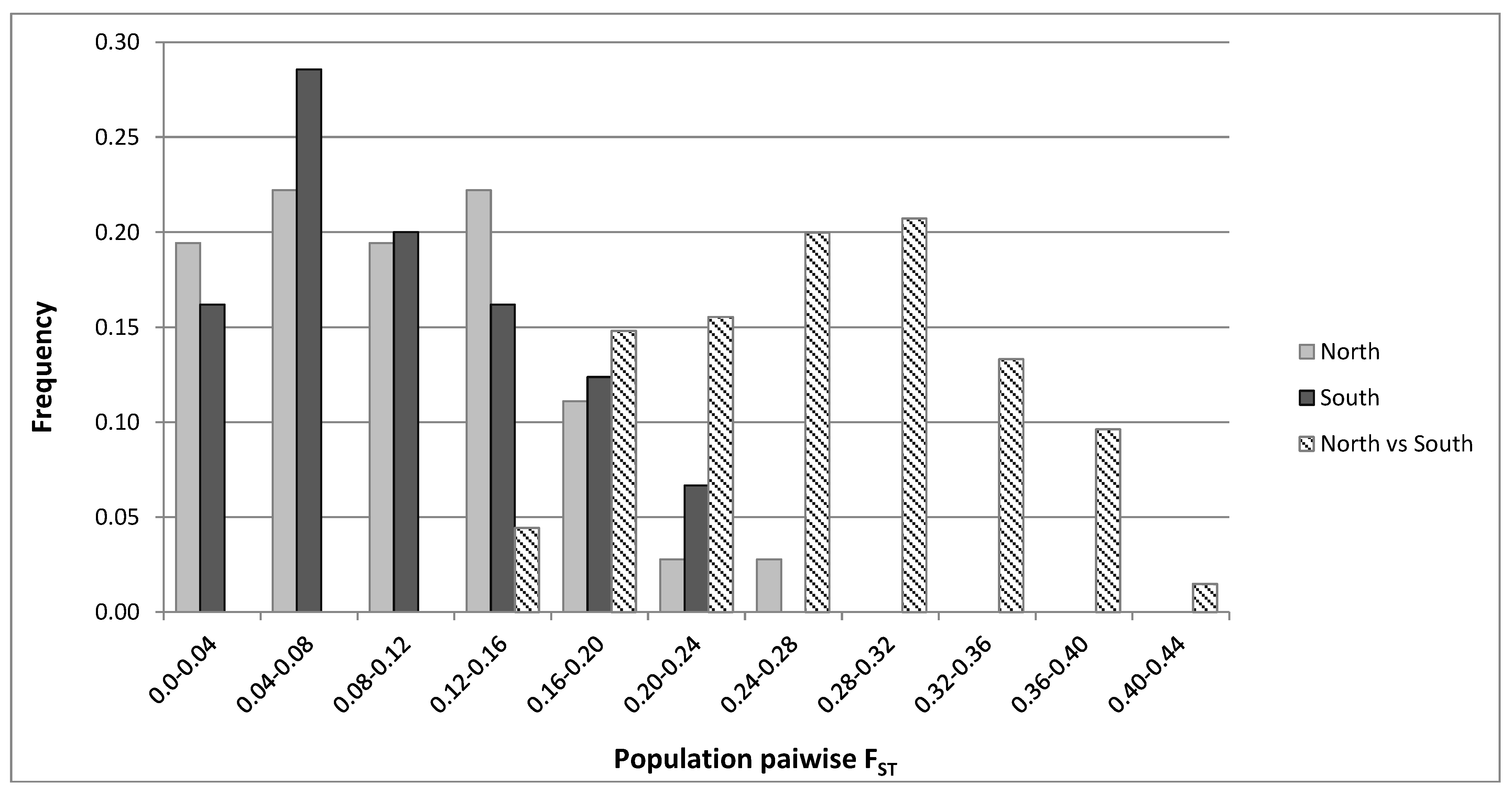

Supplement: Figure S2 — Histograms showing the distribution of pairwise F ST between populations within the northern and southern L. montandoni groups and between groups. (TIF) [file pone.0097431.s002.tif]

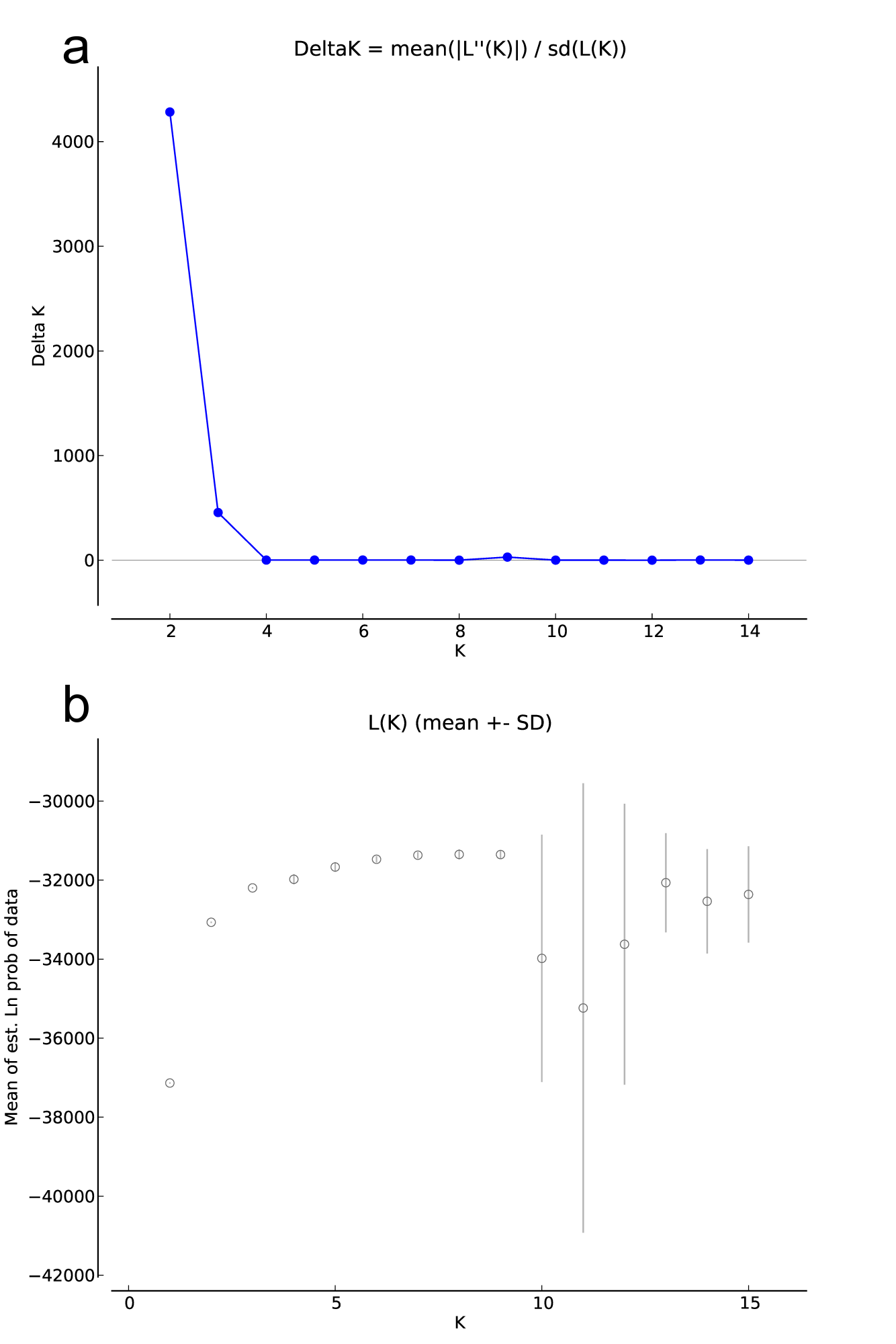

Supplement: Figure S3 — Identification of the number of groups ( K ) in Structure analysis for L. montandoni . (a) Evanno et al. (2005) method; (b) means and standard deviations (SD) of the ln-likelihood of the probability of data for various values of K. (TIF) [file pone.0097431.s003.tif]

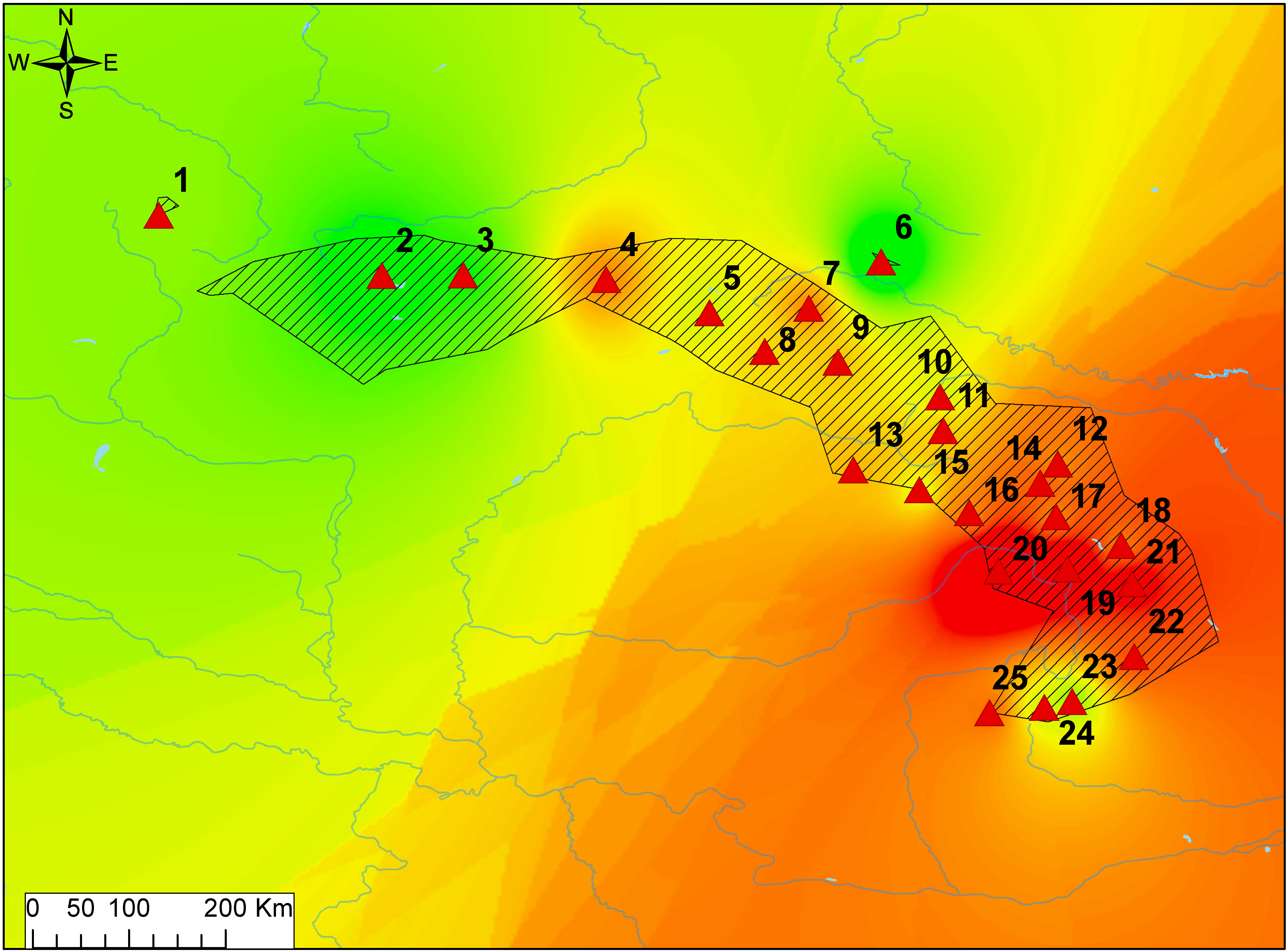

Supplement: Figure S4 — Interpolated geographic gradients of allelic richness in L. montandoni . (TIF) [file pone.0097431.s004.tif]

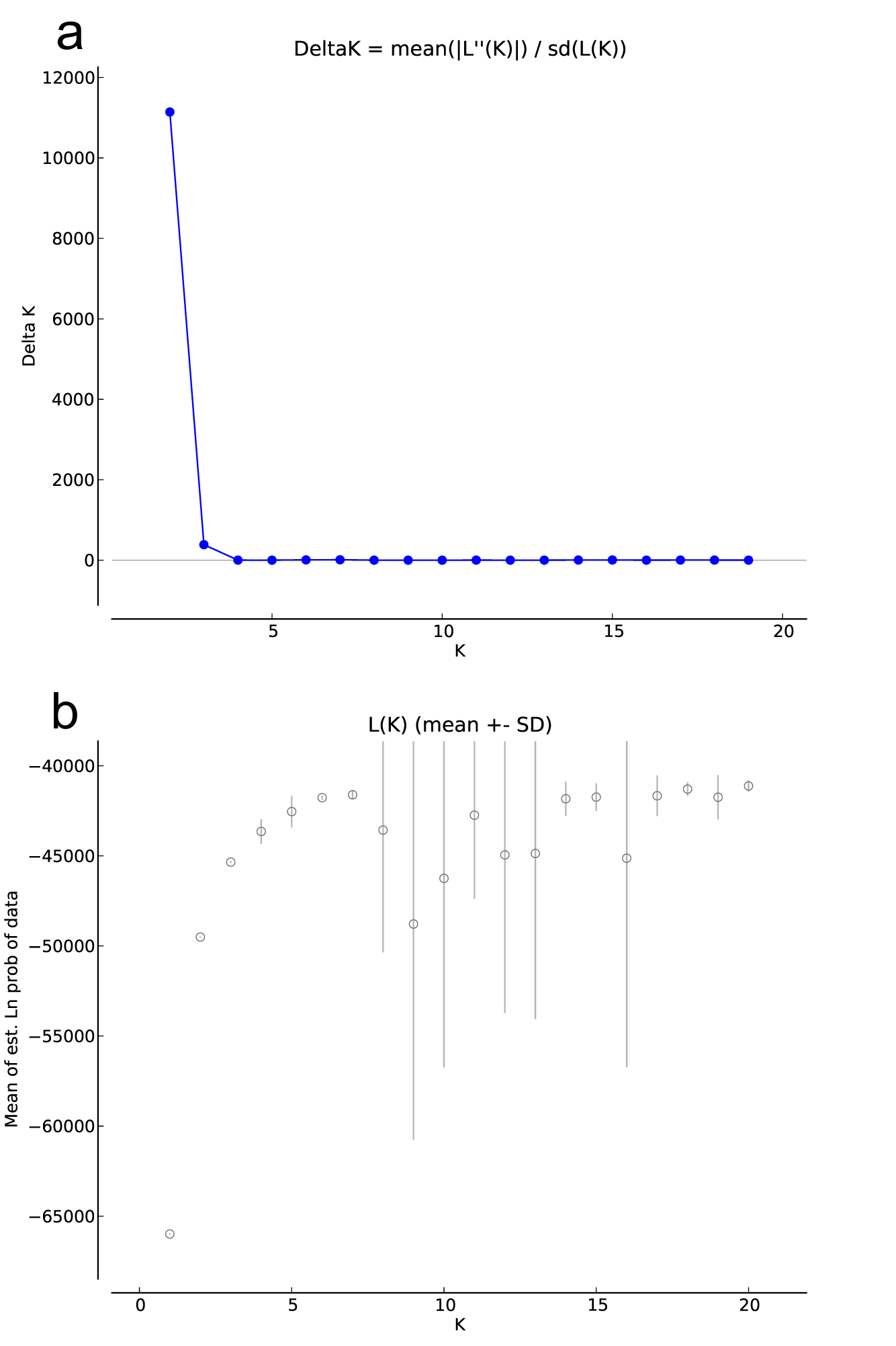

Supplement: Figure S6 — Identification of the number of groups ( K ) in Structure analysis for L. montandoni and L. vulgaris . (a) Evanno et al. (2005) method; (b) means and standard deviations (SD) of the ln-likelihood of the probability of data for various values of K. (TIF) [file pone.0097431.s006.tif]
