# Supplementary material for: Correction: Correction: Single Nucleotide Polymorphisms Reveal Genetic Structuring of the Carpathian Newt and Provide Evidence of Interspecific Gene Flow in the Nuclear Genome
Source: PLoS One. 2014 Aug 4;9(8):e105029. doi: 10.1371/journal.pone.0105029 (PMC4121281; doi:10.1371/journal.pone.0105029)
Supplement: File S2 — Republished, corrected article. (PDF) [file pone.0105029.s001.pdf]

PLOS ONE

Single Nucleotide Polymorphisms Reveal Genetic Structuring of the Carpathian Newt and Provide Evidence of Interspecific Gene Flow in the Nuclear Genome

Piotr Zieliński[1]*, Katarzyna Dudek[1], Michał Tadeusz Stuglik[1], Marcin Liana[2], Wiesław Babik[1]

1 Institute of Environmental Sciences, Jagiellonian University, Kraków, Poland, 2 Department of Comparative Anatomy, Institute of Zoology, Jagiellonian University, Krakow, Poland

Abstract

Genetic variation within species is commonly structured in a hierarchical manner which may result from superimposition of processes acting at different spatial and temporal scales. In organisms of limited dispersal ability, signatures of past subdivision are detectable for a long time. Studies of contemporary genetic structure in such taxa inform about the history of isolation, range changes and local admixture resulting from geographically restricted hybridization with related species. Here we use a set of 139 transcriptome-derived, unlinked nuclear single nucleotide polymorphisms (SNP) to assess the genetic structure of the Carpathian newt (*Lissotriton montandoni*, *Lm*) and introgression from its congener, the smooth newt (*L. vulgaris*, *Lv*). Two substantially differentiated groups of *Lm* populations likely originated from separate refugia, both located in the Eastern Carpathians. The colonization of the present range in north-western and south-western directions was accompanied by a modest loss of variation; admixture between the two groups has occurred in the middle of the Eastern Carpathians. Local, apparently recent introgression of *Lv* alleles into several *Lm* populations was detected, demonstrating increased power for admixture detection in comparison to a previous study based on a limited number of microsatellite markers. The level of introgression was higher in *Lm* populations classified as admixed than in syntopic populations. We discuss the possible causes and propose further tests to distinguish between alternatives. Several outlier loci were identified in tests of interspecific differentiation, suggesting genomic heterogeneity of gene flow between species.

Citation: Zieliński P, Dudek K, Stuglik MT, Liana M, Babik W (2014) Single Nucleotide Polymorphisms Reveal Genetic Structuring of the Carpathian Newt and Provide Evidence of Interspecific Gene Flow in the Nuclear Genome. PLoS ONE 9(5): e97431. doi:10.1371/journal.pone.0097431

Editor: Daniele Canestrelli, Tuscia University, Italy

Received February 18, 2014; **Accepted** April 19, 2014; **Published** May 12, 2014

Copyright: © 2014 Zieliński et al. This is an open-access article distributed under the terms of the Creative Commons Attribution License, which permits unrestricted use, distribution, and reproduction in any medium, provided the original author and source are credited.

Funding: The work was supported by the Polish National Science Center grant nr 8171/B/P01/2011/40 to WB and by the Jagiellonian University grant DS/WBiNoZ/INoS/762/13. MTS is the recipient of DOCTUS stipend. The GoldenGate Genotyping (Illumina) was performed in Genome Analysis Laboratory (Laboratory of High Throughput Technologies, IBMiB, Faculty of Biology UAM, Poznan), funded by National Multidisciplinary Laboratory of Functional Nanomaterials NanoFun nr POIG.02.02.00-00-025/09 (Innovative Economy Operational Programme, Priority Axis 2: R&D Infrastructure, Action 2.2: Support of Formation of Common Research Infrastructure of Scientific Units). The funders had no role in study design, data collection and analysis, decision to publish, or preparation of the manuscript.

Competing Interests: The authors have declared that no competing interests exist.

* E-mail: piotr.zielinski@uj.edu.pl

Introduction

Most species are genetically structured, and genetic structure is often observed at multiple spatial scales [1,2]. Genetic structure is the result of a complex interplay of drift, gene flow, and natural selection acting on standing variation and new mutations [3–5]. The relative importance of these evolutionary forces is contingent on biological features of the organisms [6], and has also been affected by large-scale historical events, such as the Pleistocene climatic oscillations [2,7]. Identification of factors responsible for the observed spatial structuring of genetic diversity is a major goal of population genetics [8]. The quantification and understanding of genetic structure within species are of fundamental importance for inferential studies of population history, population ecology and biodiversity conservation [3,9,10]. Analyses of genetic structure are also essential for several aspects of the study of adaptation [5,11–18].

Genetic variation within species is commonly structured in a hierarchical manner which may result from superimposition of processes acting at different spatial and temporal scales. For example the impact of major climatic oscillations is clearly visible in the patterns of genetic differentiation observed currently in temperate and boreal species [2,19]. This is believed to reflect mainly secondary contact and partial admixture of populations derived from separate refugia with a contribution of processes related to the expansion itself, such as allele surfing [4,7]. Within these major geographic groups, populations are differentiated due to limited dispersal producing isolation by distance [20,21].

In species with limited dispersal capabilities, signatures of past subdivision are detectable for a long time [2,22]. This may be due to a combination of limited dispersal per se and differential adaptation in refugia superimposed on contemporary ecological gradients [23]. Admixture may also be delayed or prevented by the accumulation of intrinsic incompatibilities between populations [24,25] and poor dispersers appear to speciate on smaller geographic scales [26]. Thus studying contemporary genetic structure in taxa characterized by limited dispersal is likely to

provide ample information about historical and demographic events. Amphibians and in particular salamanders are ideal for such inferences [27,28]. Another advantage of such taxa is that they retain historical information about spatial variation of genetic exchange with related, incompletely reproductively isolated species ([29] and references therein).

Detecting, quantifying and interpreting genetic structure requires appropriate tools. Single nucleotide polymorphisms (SNPs) are powerful markers well suited for assessing genetic structure [30,31]. They are amendable to high throughput, cost-effective and reliable genotyping through array-based [32] and genotyping by sequencing [33] approaches. If SNP discovery and genotyping are performed separately, the researcher has control over the location and other characteristics of the SNPs selected for genotyping. For instance, if polymorphism data from transcriptome sequencing are available, SNPs located in known protein coding genes can be selected for genotyping, providing information about variation in functionally important regions. On the other hand, a discover-then-genotype approach introduces ascertainment bias, which distorts the picture of variation obtained from a larger sample [34]. However, initial discovery of SNPs in a smaller sample may be desirable for some applications, such as detection and quantification of population structure [35,36]. This is because the discovery process is biased towards more variable SNPs, thus increasing the per-marker information content, especially if SNP discovery is performed in a random or geographically diverse sample [37]. A distinct advantage of SNPs over microsatellites is that orders of magnitude more locations in the genome, can be easily interrogated. Thus SNPs offer a truly genome-wide perspective, essential if the biological processes of interest affect portions of the genome differentially [38–40].

Here we investigate the genetic structure of populations of the Carpathian newt (*Lissotriton montandoni*, *Lm*), a species which has apparently survived the glacial period in the Carpathians [41], an important refugial area [42,43]. Two processes appear to have profoundly affected this species and shaped the genetic structure currently observed. The first includes climatic oscillations during the Pleistocene, likely responsible for the observed regional-scale genetic structuring. The second involves hybridization with and introgression from its widely distributed congener, the smooth newt (*L. vulgaris*, *Lv*). Previous studies [29,41,44,45] demonstrated that *Lm* is genetically differentiated across its range in both mitochondrial and nuclear (microsatellites) genome. Patterns of genetic differentiation and species distribution modeling performed by Zieliński et al. [29] suggest several glacial refugia in the Carpathians. While multiple, spatially and temporally distinct introgression events from *Lv* resulted in complete mtDNA replacement in *Lm*, very little recent interspecific nuclear gene flow was suggested by microsatellite markers [29]. However, interspecific gene flow in some parts of the nuclear genome has been extensive, as evidenced by data from the Major Histocompatibility Complex genes [45].

A set of transcriptome-derived SNPs and extensive sampling are used herein to address the following issues. First, we compare genetic structure inferred from the genome-wide sample of SNP markers with that estimated previously [29] from a much smaller number of microsatellites. Specifically we wanted to determine the number of genetic clusters (which may correspond to glacial refugia) supported by SNP markers, delineate their distribution and estimate the extent of admixture between them. To this end we use a comprehensive, uniform sampling, including previously undersampled Ukrainian Carpathians where admixture between genetic clusters was expected. Second, we test whether introgression from *Lv* is detectable in the nuclear genome of *Lm* with an increased number of markers, and if so, whether introgression varies geographically. Populations in which both species co-occur were also sampled across the range to estimate the admixture in syntopy. Third, we apply outlier analysis to test heterogeneity of gene flow within and between species and identify genes departing from the genomic average; such genes may be involved in population or species-specific adaptations.

Material and Methods

Sample collection

Altogether we analyzed 473 individuals from 40 populations: 25 populations of *Lm* (298 individuals), 7 syntopic populations in which both species co-occur (83 individuals) and 8 populations of *Lv* (92 individuals) (Fig. 1; Table 1). Sampling sites were selected to cover the *Lm* range uniformly and to reflect *Lv* diversity in the surrounding areas. The average per population sample size, 12, might be considered low for some of the population genetic analyses, however we decided to rather maximize the number of markers as it was shown that this might be beneficial for robust landscape genetic inferences [46]. Throughout the text, we use terms population or locality interchangeably to refer to a particular breeding site consisting of one or more closely located water bodies. Adult newts were sampled by dip-netting during breeding season. Animals were released after tailtips were collected. Tissue samples were stored in 95% ethanol until DNA extraction. DNA was extracted using the Wizard Genomic DNA Purification Kit (Promega).

Ethics statement

Animal samples were collected under permits: DOPozgiz-4200/II-78/3702/10/JRO provided by the Polish General Director for Environmental Protection, 03.04.12 No. 67 provided by the National Academy of Sciences of Ukraine and 3256/9.07.2010 provided by the Romanian Commission for Protection of Natural Monuments. Samples were collected with institutional animal ethics approval (number 101/2009), issued by the First Local Ethical Committee on Animal Testing at the Jagiellonian University in Krakow. Tissue samples were collected according to the requirements of these institutions: adult newts were captured by dip netting and tissue samples from tail tips were taken under anesthesia. Immediately after recovery from anesthesia, newts were released at the collection site. The sampling locations were not privately owned or protected in any way.

SNP discovery, assay development, and genotyping

SNPs were identified in liver transcriptomes of six *Lm* individuals sampled to encompass the genetic diversity of the species (Fig. 1). Transcriptomes were sequenced using Illumina technology and de novo assembled with Trinity [47]. Details of transcriptome sequencing and assembly will be provided elsewhere (Stuglik et al. in prep). We used a custom bioinformatic pipeline [48] to construct transcriptome-based gene models (TGM) from the Trinity output. Reads were mapped to this reference transcriptome with Bowtie2 [49] and SNPs were called with SAMTools 0.1.18. [50]. Next, we used blast searches against *Xenopus tropicalis* transcripts to identify TGM representing protein coding genes. To be included in the design of the genotyping assay, the SNP had to fulfill the following criteria: i) occur in a TGM which produced an unambiguous hit to a single *Xenopus* gene and to not exhibit high similarity to other TGMs in the newt reference transcriptome; this criterion was applied to minimize the incidence of false "SNPs" derived from paralogous regions; ii) have a minimum sequencing depth of 15 x and minimum

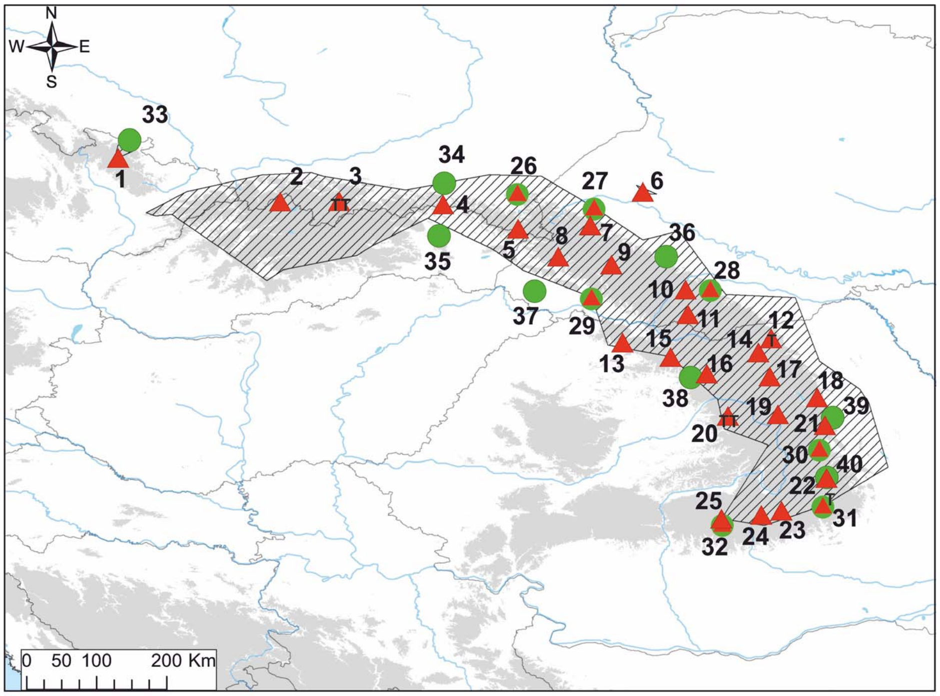

Figure 1. The distribution of sampling localities (details in Table 1). Red triangles — Lissotriton montandoni (Lm); green circles— L. vulgaris (Lv); two symbols superimposed — syntopic locality where both species co-occur; T – localities from which six Lm individuals were sampled for liver transcriptomes. The distribution of Lm (Zavadil et al. 2003 and own unpublished data) is hatched. Areas above 500 m a.s.l. are shaded.
doi:10.1371/journal.pone.0097431.g001

genotype quality of 30 phred; iii) be located at least 60 but not more than 1000 bp from the exon boundary; the latter filter was used because last exons of many genes are long and consist mostly of 3′ untranslated regions (UTR) which are poorly conserved between species; thus the length of such exons in the newt could not be reliably determined and particularly long last "exons" may be artifacts of misassembly [51]. Filtering was performed with a custom Python script. A total of 251 SNPs and their flanking sequences were scored with Illumina Assay Design Tool (ADT) and the Illumina VeraCode GoldenGate Assay was designed for 192 best scoring SNPs. GoldenGate provides codominant genotype data for polymorphic positions with two segregating variants [32]. Genotyping, primary visualization, quality assessment and filtering were performed with Illumina GenomeStudio Data Analysis Software. All loci with cluster separation score and gen train score lower than 0.2 and 0.7, respectively, were excluded from further analysis. We also excluded loci with minor allele frequency (MAF)<1% and less than 90% genotyped individuals.

Population genetics analyses

Allele frequencies for each locus, tests of Hardy–Weinberg equilibrium and tests of linkage disequilibrium (LD) were calculated in GENEPOP 4.1.2 [52]; the type I error was controlled using the false discovery rate (FDR) approach implemented in QVALUE 1.0 [53,54]. Expected heterozygosity (H_E), was calculated in the R package adegenet [55]. Allelic richness (*AR*) was calculated in FSTAT 2.9.3.2 [56]. We interpolated geographic gradients in H_E and *AR* using inverse distance weighting (IDW) in ArcGIS v 10.0 (ESRI, Redlands, CA, USA). Pairwise F_{ST} values between populations and their significance were computed in Arlequin 3.5 [57]. Multidimensional scaling (MDS) was used for visualization of the F_{ST} matrix. Principal component analysis (PCA) was performed in R using adegenet. The significance of correlations between genetic and geographical distances was calculated using the Mantel test implemented in IBDWS [58]. A population tree was constructed in POPTREE2 [59] from the pairwise F_{ST} [60] matrix using the neighbor-joining method. The number of genetic clusters was determined and assignment of individuals to clusters was performed using the Bayesian approach implemented in STRUCTURE 2.3.3 [61–63]. We ran Structure on two separate datasets: *Lm*, which included only morphologically pure *Lm* populations and *Lm&Lv* comprising *Lm*, *Lv* and syntopic populations. We ran 10 analyses for each *K* 1-15 for *Lm* and 10 replicate runs for each *K* 1–20 for *Lm*&*Lv* dataset. In each case, the admixture model was applied and the runs consisted of 250 000 MCMC burnin steps followed by 1 000 000 post-burnin iterations. We performed inferences under the model of correlated allele frequencies for *Lm*, whereas the uncorrelated model was used for *Lm*&*Lv* dataset, because *Lm* and *Lv* populations were expected to be more divergent on average. To determine the most likely number of genetic clusters supported by the data, we calculated ΔK, a measure of second order rate of change in the likelihood of data [64], using the online software Structure Harvester [65]. Analysis of molecular variance (AMOVA) in Arlequin was used to partition SNP variation into hierarchical levels. Two groupings of populations were used: i) suggested by Structure; ii) supported by the methods based on genetic distances between populations. Significance levels for variance components were estimated using 10 000 permutations.

To identify markers departing significantly from the genome-wide average of differentiation among populations a scan for F_{ST} outliers was performed. In order to minimize the number of false positives the outlier detection was performed under a hierarchical island model [66] in Arlequin. We performed separate scans for differentiation within *Lm* and for interspecific differentiation. In

Table 1. Sampling.

Number	Locality	Country	N individuals	Species	Longitude E	Latitude N
1	Jeseniki	CZ	12	*Lm*	17.31	50.07
2	Sihelne	SK	12	*Lm*	19.39	49.51
3	Łopuszna	PL	14	*Lm*	20.14	49.51
4	Krempna	PL	12	*Lm*	21.48	49.47
5	Smerek	PL	12	*Lm*	22.44	49.16
6	Rakovets	UA	12	*Lm*	24.04	49.63
7	Pereprostynya	UA	12	*Lm*	23.36	49.21
8	Zbyny	UA	12	*Lm*	22.95	48.80
9	Vyshkivsky Pass	UA	12	*Lm*	23.63	48.70
10	Mykulychyn	UA	12	*Lm*	24.59	48.38
11	Dzembronia	UA	9	*Lm*	24.62	48.07
12	Suceviţa	RO	12	*Lm*	25.68	47.75
13	Pasul Gutâi	RO	12	*Lm*	23.78	47.70
14	Pasul Pascanu	RO	12	*Lm*	25.52	47.57
15	Romuli	RO	12	*Lm*	24.39	47.51
16	Lunca Leşului	RO	12	*Lm*	24.86	47.30
17	Holda	RO	12	*Lm*	25.67	47.27
18	Cuejdiu	RO	12	*Lm*	26.27	47.00
19	Lacu Roşu	RO	12	*Lm*	25.77	46.78
20	Brădeţelu	RO	12	*Lm*	25.13	46.76
21	Bolătău	RO	12	*Lm*	26.38	46.64
22	Pasul Musat 1	RO	12	*Lm*	26.40	45.96
23	Săcele	RO	12	*Lm*	25.82	45.55
24	Predeal	RO	11	*Lm*	25.56	45.49
25	Voina	RO	12	*Lm*	25.05	45.44
26	Kuźmina	PL	11	*Lm&Lv*	22.43	49.62
27	Bronytsya 1	UA	12	*Lm&Lv*	23.41	49.43
28	Lyucha	UA	12	*Lm&Lv*	24.91	48.38
29	Lypcha	UA	12	*Lm&Lv*	23.38	48.28
30	Valea Uzului	RO	12	*Lm&Lv*	26.30	46.34
31	Penteleu	RO	12	*Lm&Lv*	26.35	45.61
32	Lereşti	RO	12	*Lm&Lv*	25.06	45.37
33	Pokrzywna	PL	11	*Lvv*	17.45	50.29
34	Jasło	PL	12	*Lvv*	21.49	49.74
35	Lipníky	SK	13	*Lvv*	21.42	49.07
36	Rosilna	UA	8	*Lvv*	24.34	48.80
37	Dertsen	UA	12	*Lva*	22.65	48.35
38	Strâmba	RO	12	*Lvv*	24.65	47.25
39	Tazlău	RO	12	*Lvv*	26.47	46.73
40	Pasul Musat 2	RO	12	*Lvv*	26.40	45.97

Country abbreviations: CZ – Czech Republic, PL – Poland, RO – Romania, SK – Slovakia, UA – Ukraine. Species and subspecies abbreviations: *Lm* – *Lissotriton montandoni*, *Lva* – *L. vulgaris ampelensis*, *Lvv* – *L. v. vulgaris*, *Lm&Lv* – syntopic populations.
doi:10.1371/journal.pone.0097431.t001

each case 50 000 coalescent simulations with 2 groups of 100 demes were performed to obtain the null distribution of *F*-statistics. We selected candidate loci based on F_{ST} or F_{CT} values falling into the 1% upper and lower quantile as suggested by Excoffier et al. [67]. F_{CT} allows for identification of outlier loci between the groups of populations whereas F_{ST} identifies outlier loci among populations after accounting for higher-level structure [67]. Genes containing outliers were annotated by similarity blastx search against the nr protein database.

Results

Variation

Genotyping was successful for 139 out of 192 markers (72%) and these were used for population genetic analyses (Table S1)

(DRYAD entry: doi:10.5061/dryad.211ck). The proportion of missing data among 473 genotyped individuals was very low (< 0.2% single-locus genotypes). All 139 markers were polymorphic in *Lm* and 112 (81.6%) in *Lv* (Table S1). No significant deviations from Hardy–Weinberg expectations were detected at the FDR 0.05 which indicates that null alleles are very rare in our markers (Table S2). Tests for linkage disequilibrium across populations detected significant LD at the FDR 0.05 for 12 pairs of loci, however significant results were found only in three syntopic populations. Thus significant LD resulted not from physical linkage but from local admixture, and we consider all markers as segregating independently.

The expected heterozygosity (H_E) ranged from 0.05 (locality 33, *Lv*) to 0.29 (locality 29, syntopic) with a mean of 0.19 (SD = 0.07). H_E was significantly higher in *Lm* than *Lv* (*U*-test, $P = 2.9\times10^{-5}$) most likely due to ascertainment bias, as only markers known to be polymorphic in *Lm* were assayed. Within *Lm* H_E was lowest in population 6, isolated in the Ukrainian Podolian Upland and highest in population 20 located in the Romanian Transylvanian Plateau, and ranged from 0.19 to 0.27 respectively, with a mean of 0.23 (SD = 0.01) (Fig. S1). Within *Lv* H_E ranged from 0.05 in the northernmost locality 33 to 0.10 in locality 36 with a mean of 0.08 (SD = 0.01). H_E in syntopic populations spanned a broad range from very low (0.06, locality 31) to the highest overall (0.29, locality 29), most likely depending on the frequency of both species in the population (Fig. S1b).

Genetic structure and diversity in *Lissotriton montandoni*

Genetic differentiation between *Lm* populations varied from negligible (F_{ST}<0.001 $P = 0.39$ between 14 and 17 located in the northern part of the Romanian Carpathians), to strong ($F_{ST} = 0.408$ $P<10^{-3}$ between populations 1 and 24 at the opposite limits of the species distribution) (Table S3). The MDS plot of pairwise F_{ST} revealed two major, genetically differentiated groups of populations with distinct geographic distributions in the northern and southern part of the species range (Fig. 2a). Pairwise F_{ST} values within groups were similar (averages of 0.100 and 0.098 in the northern and southern group, respectively), and overlapped only slightly with the distribution of pairwise F_{ST} between populations from different groups (mean 0.271; randomization test $P<0.001$; Fig. S2). Within the northern group two populations appeared distinct from the rest. Notably both are isolated from the continuous part of the species range (Fig. 1). The westernmost locality 1 in the Sudetes Mountains is separated from the main range by the Moravian Gate and locality 6 in the Ukrainian Podolian Upland by the Dniester river. The two groups of *Lm* populations are strongly supported also by the population tree (Fig. 3).

The presence of two genetic clusters is also evident from individual-based analyses.

In principal component analysis (PCA) PC1 (15.8% of variance explained) separated newts from northern and southern populations (Fig. 2b). The Evanno [64] method also supported $K = 2$ as the most likely number of clusters in the Structure analysis (Fig. S3). Structure detected some admixture between the two clusters in the Ukrainian Carpathians and the northern part of the Romanian Carpathians. Admixture was strongest in population 10 which was therefore excluded from the AMOVA analysis (Fig. 4). AMOVA attributed 19.5% of total variation to differentiation between clusters and 8.2% to differentiation between populations within clusters (Table 2). Whereas no alleles were private to any population, 8 and 20 alleles were private for the northern and southern group, respectively. No significant differences between groups were detected in H_E (*U*-test, $P = 0.37$), but allelic richness

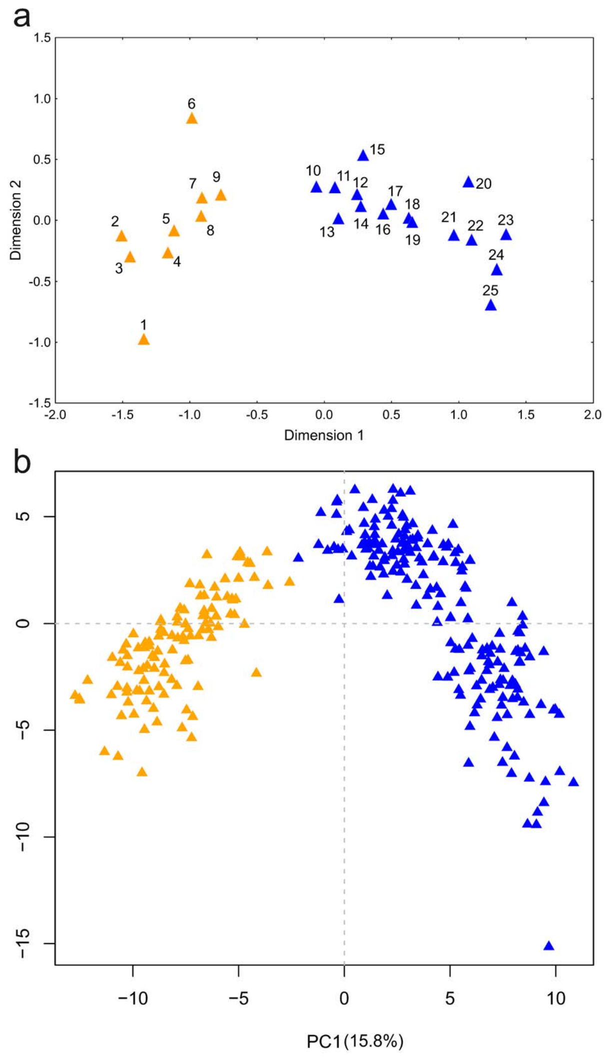

Figure 2. Genetic differentiation within *L. montandoni*. (a) Non-metric two-dimensional scaling of the pairwise F_{ST} matrix; orange – populations from the northern group; blue – populations from the southern group; (b) Principal Component Analysis (PCA) performed on individual genotypes; in parentheses percentage of variance explained by principal components; orange – individuals from the northern group; blue – individuals from the southern group.
doi:10.1371/journal.pone.0097431.g002

was higher in the southern group (1.62 vs, 1.57; *U*-test, $P = 0.0042$) (Fig. S4). As could have been expected from the significant among-population differentiation, there was a strong, highly significant isolation by distance observed both at the level of the entire species (Mantel test, $r = 0.89$, $P<0.001$) and within genetic clusters (North: $r = 0.78$, $P<0.001$; South: $r = 0.78$, $P<0.001$) (Fig. S5).

Differentiation within *Lissotriton vulgaris*

Genetic structure among *Lv* populations around the Carpathians is stronger and presumably deeper than that within *Lm*. A deep split between populations outside the Carpathian belt and those in the Carpathian Basin is visible in the population tree (Fig. 3), MDS (Fig. 5a) and PCA plot (PC3, Fig. 5c). Structuring within the Carpathian Basin is also pronounced, as substantial genetic distance separates the single analysed population of *L. vulgaris ampelensis* (locality 38) from two populations of the nominal subspecies *L. vulgaris vulgaris* (Fig. 3, 5a, 5c).

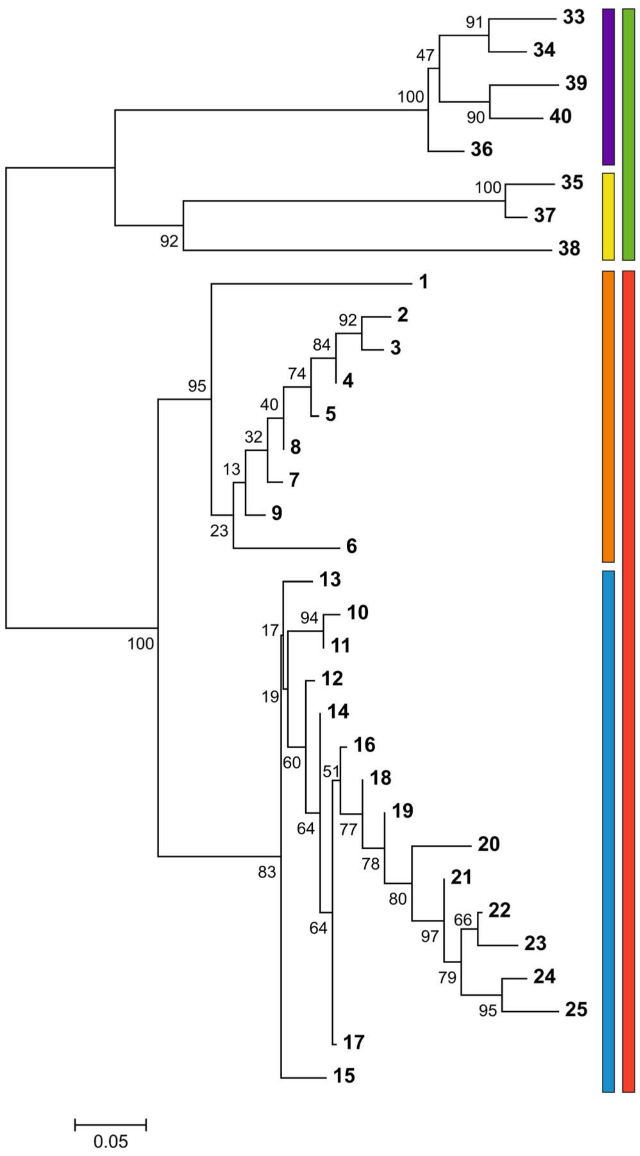

Figure 3. Relationships among populations. A neighbor-joining tree was constructed from the matrix of pairwise F_{ST}; syntopic populations were excluded. Robustness of relationships was tested with 1000 bootstrap replicates. Red – *L. montandoni*: orange – northern, blue – southern group; green – *L. vulgaris*: yellow – populations in the Carpathian Basin, violet – populations outside the Carpathian Basin.
doi:10.1371/journal.pone.0097431.g003

Genetic differentiation and gene flow between species

Strong differentiation between *Lm* and *Lv* was detected by the population-based analyses (Table S3, Fig. 3, 5a). AMOVA revealed that 43.5% variation was distributed between species and 13.3% between populations within species (Table 2). Three of seven syntopic populations occupied intermediate positions in the MDS plot (Fig. 5a). However, an overwhelming majority of newts in syntopic populations fell within the range of variation of either one or the other species; only a handful of substantially admixed individuals and possibly a single F1 hybrid were detected (Fig. 5b, 6). $K = 2$ was strongly supported by Structure when the two species were analyzed together (Fig. 6, S6). Structure confirmed that in syntopic populations admixture is limited, genotypes of two parental species dominate and significantly admixed individuals are rare. Structure also provided an important insight which was not visible in PCA results: a clearly detectable (>3%) admixture of *Lv* genes in four *Lm* populations. Three of these were in the southern part of the Romanian Eastern Carpathians (localities 19, 20, 25) and one was the isolated locality 1 in the Sudetes Mountains; the average admixture in these populations was 8.5%. No admixture of *Lm* genes was detected in *Lv* populations. The comparison of the average proportion of admixture on both genetic backgrounds in syntopic populations demonstrated that the mean admixture was very low, ca. 2% and that the proportion of admixture did not differ (U-test, $P = 0.24$) between *Lv* and *Lm* backgrounds.

Detection of outliers

The scan for outliers performed in *Lm* (locality 10 excluded, see above) revealed 14 F_{ST} outliers (10.0%) at the significance level of 0.01: 9 loci (9, 17, 25, 28, 34, 45, 57, 73, 137) showed an excess of differentiation among populations (candidates for local adaptations) and 5 (4, 6, 22, 84, 93) loci were less differentiated than expected under neutrality (candidates for balancing selection) (Fig. 7a). Three F_{CT} outliers (45,73,75) were identified as candidates for diversifying selection between the northern and southern *Lm* groups and two (72, 89) as candidates for balancing selection (Fig. 7b).

Screening for outlier loci between *Lm* and *Lv* was performed excluding syntopic populations (26–32). A total of nine (6.5%) F_{CT} outlier loci were identified: eight (15, 72, 79, 112, 116, 117, 128, 129) were more differentiated than expected under the neutral model and one (126) was less differentiated (Fig. 7c). Only one of the interspecific outliers was a nonsynonymous polymorphism. Locus 72 located within gene *SRSF1*, involved in splicing regulation, was classified as a candidate for balancing selection between the northern and southern *Lm* groups and at the same time as a candidate for divergent selection between species.

Discussion

Isolation in glacial refugia and limited dispersal determine the genetic structure of *Lm*

Two clearly differentiated genetic units were identified in *Lm* by SNP data: the northern group in the Western Carpathians and the western part of the Eastern Carpathians, and the southern group across the rest of the species range. Admixture between them occurs around the Romanian-Ukrainian border. Zieliński et al. [29] identified three units in microsatellite data: our southern group combines their eastern and southern units. Can the discrepancy between the two studies be reconciled? Below we argue that SNPs reflect the history and differentiation better than microsatellites and offer several explanations of the apparent discrepancy between the datasets. We consider the alternative explanation that SNP data fail to detect true differentiation unlikely on several grounds. First, an overwhelming majority of pairwise F_{ST} values calculated from SNPs were significant, demonstrating substantial power to detect differentiation. Second, remarkably strong isolation by distance is observed in *Lm* and the apparent break between the eastern and southern groups of Zieliński et al. [29] coincides with a gap in their sampling. This gap has been filled by the present study. Thus, isolation by distance and non-uniform sampling may have resulted in delineation of the apparently distinct unit [68,69]. Third, the population tree based on SNPs shows a remarkable pattern consistent with colonization from two refugia, but it is difficult to explain under the assumption of expansion from three refugia. While in both groups relationships between some populations are

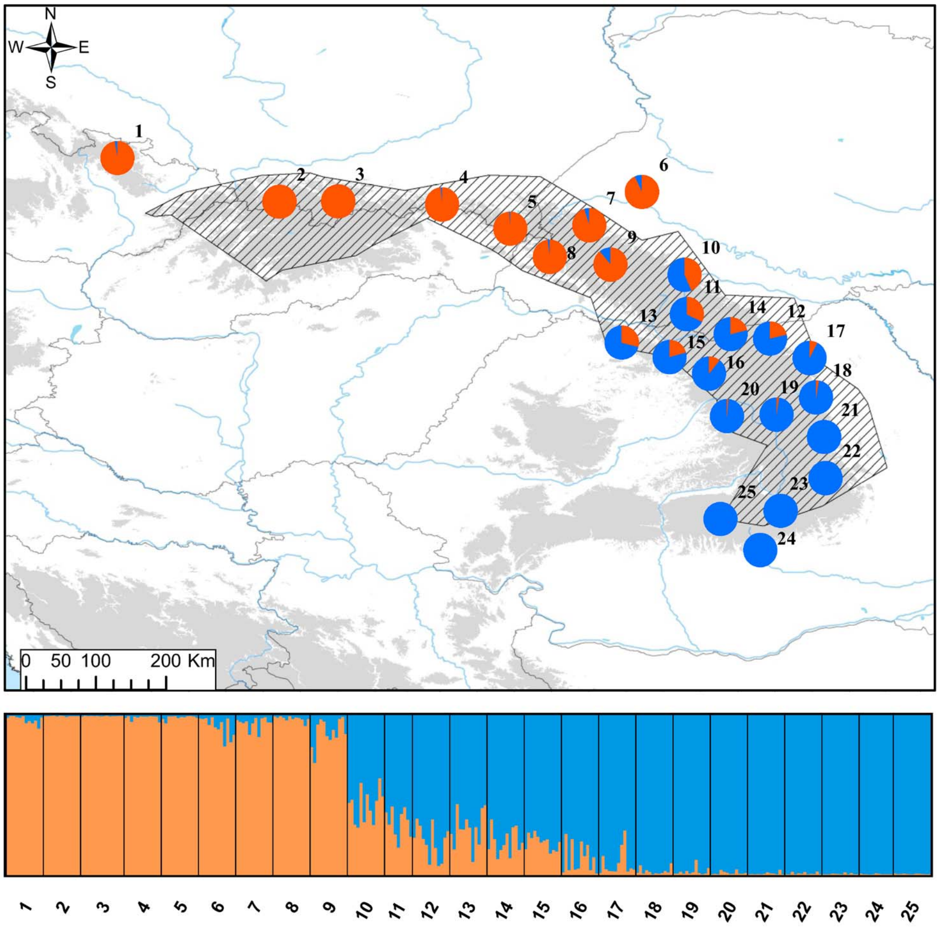

Figure 4. Genetic structure of *L. montandoni* inferred by Structure for $K=2$ groups. For each population pie charts show the fraction of the genes from the northern (orange) and southern (blue) groups.
doi:10.1371/journal.pone.0097431.g004

poorly resolved, in each group several populations are related in a nested fashion, with progressively longer branches at the deeper nesting levels. Nesting involves populations distributed from the center to the periphery of the species range – to the west in the Western Carpathians and to the south-west in the Romanian Carpathians. We hypothesize that the populations with poorly resolved relationships are those inhabiting the refugial areas and sharing most variation retained there. Populations related to each other in a nested fashion would be those which colonized the present range through serial events [4,70]. Taking the evidence together, we propose that the location of the refugium for the northern group was in the Eastern Carpathians close to the Polish-Ukrainian border, and that the refugium for the southern group was in the central part of the Eastern Carpathians in Romania. Species distribution models for the LGM reported by Zieliński et al. [29] are broadly consistent with the proposed location of

Table 2. Results of the Analysis of Molecular variance (AMOVA).

Source of variation	d.f.	Sum of squares	Percentage of variation explained	p
Two groups within L. montandoni				
Among groups	1	1260.054	19.52	<0.0001
Among populations within groups	22	1330.68	8.16	<0.0001
Within populations	548	8988.36	72.32	<0.0001
Two species				
Among groups	1	4019.136	43.47	<0.0001
Among populations within groups	31	3545.323	13.31	<0.0001
Within populations	747	10316.708	43.22	<0.0001

AMOVAs were performed for: i) two groups within *L. montandoni*; ii) two species (excluding syntopic populations).
doi:10.1371/journal.pone.0097431.t002

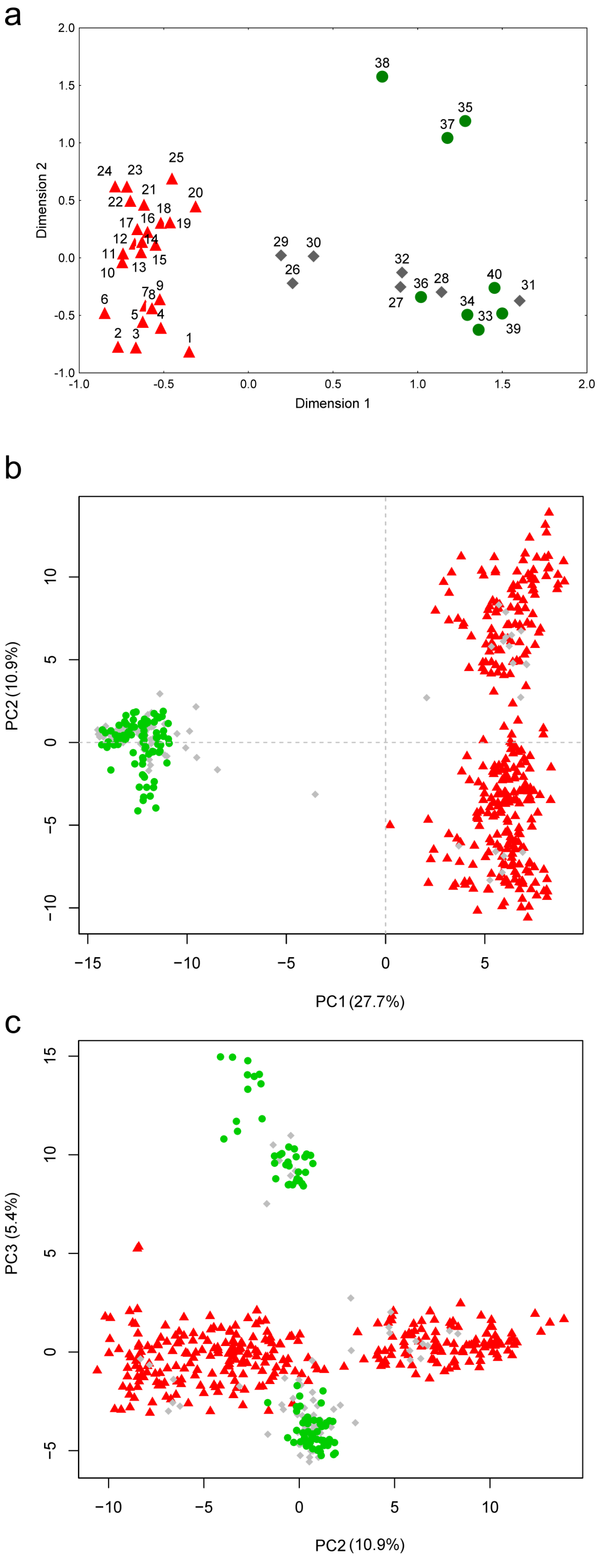

Figure 5. Genetic differentiation between *L. montandoni* (*Lm*) and *L. vulgaris* (*Lv*). (a) Non-metric two-dimensional scaling of the matrix of pairwise F_{ST} between populations; red triangles – *Lm*; green circles – *Lv*; grey diamonds – syntopic populations; (b) and (c) Principal Component Analysis (PCA) performed on individual genotypes; in parentheses percentage of variance explained by principal components; red triangles – *Lm*; green circles – *Lv*; grey diamonds – individuals from syntopic populations.
doi:10.1371/journal.pone.0097431.g005

refugia. The role of the Carpathians as a major refugium for European biota has recently been well documented [42,43,71,72]. Multiple species show genetic differentiation between the Western, Eastern and Southern Carpathians, pointing to the presence of several refugia (reviewed in [29,72]). So far, a refugium in the western part of the Eastern Carpathians has to our knowledge not been proposed.

Expansion from refugia is commonly accompanied by loss of variation [4,73]. Reduction of genetic variation along the postulated expansion routes is visible in our data, but the signal is not strong, and may be distorted locally by introgression from *Lv* (see below). Hence expansion was apparently not accompanied by severe bottlenecks and thus only a minor fraction of variation has been lost. The strongest reduction in genetic variation occurred in locality 6 in the Podolian Upland isolated from the main portion of the range. This population is a remnant of a geographically remote group of *Lm* populations [74] which has been hypothesized to be isolated from the main part of the range for several thousand years [75].

Salamanders often exhibit low individual mobility and strong philopatry [27,76]. Genetic differentiation between salamander populations appears to reflect these features although the geographic scale of subdivision differs among species [77,78], which may be related to habitat characteristics [79,80] and to life-history traits [81]. In continuous habitats limited dispersal abilities are likely to generate isolation by distance patterns with a gradient of genetic differentiation among sites, on which larger-scale, hierarchical differentiation reflecting geographic or environmental barriers may be superimposed [80,82]. A comparison of our results with those of a study [83] on fine-scale genetic differentiation in *L. vulgaris graecus* suggests that a combination of isolation by distance, probably due to limited dispersal, and spatial clustering due to historical fragmentation and/or landscape barriers occurs in *Lissotriton* newts at both micro- and macroscales.

Local introgression of *Lv* alleles into the *Lm* nuclear genome is detectable with SNP markers

A major finding of the present study is substantial introgression of *Lv* nuclear alleles into some *Lm* populations. This is contrary to the findings of Zieliński et al. [29] who detected very little recent nuclear introgression in either direction. One likely explanation for the difference between the studies is the number of markers employed [84]. While we analyzed 139 unlinked SNPs, inference about introgression in the previous study was based on only 10 microsatellites. The observed discrepancy does not result from differences in sampling because three of four admixed populations were analysed in both studies. As SNP markers were discovered in a sample of *Lm* individuals, our study did not use diagnostic markers. This could be considered a weakness if viewed from the perspective of classical studies of hybrid zones which usually employed a limited number of diagnostic markers. However, because of the widespread genomic heterogeneity of interspecific gene flow [38,40,85,86], such diagnostic markers may constitute a highly nonrandom sample of the genome, enriched in genomic regions strongly differentiated between species. In our opinion randomly selected polymorphisms are better suited for an unbiased assessment of introgression. We acknowledge that ideally

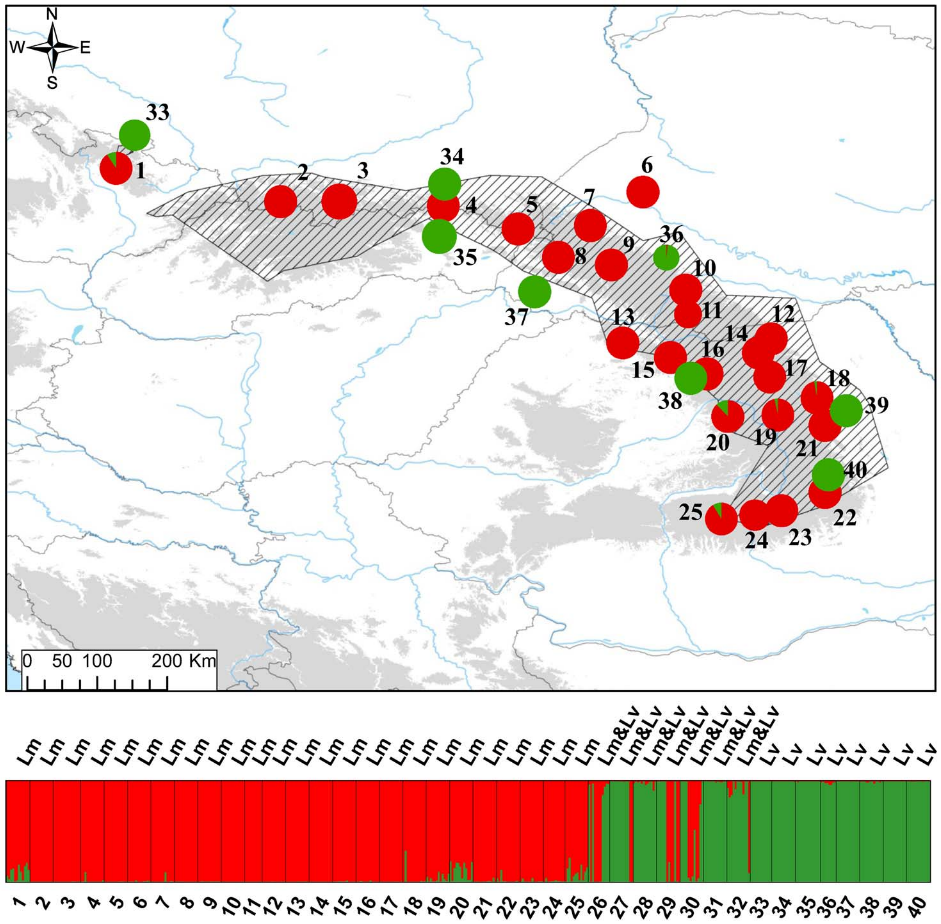

Figure 6. Genetic differentiation and admixture between *L. montandoni* and *L. vulgaris* inferred by Structure for $K=2$ groups. For each population pie charts show the fraction of *L. montandoni* (red) and *L. vulgaris* (green) genes.
doi:10.1371/journal.pone.0097431.g006

both species should be included in the discovery panel; this would however limit the number of polymorphic loci useful for the assessment of genetic structure within *Lm*. The current study demonstrates two peculiar features of *Lm* x *Lv* hybridization. First, appreciable (>3%) introgression was detectable only locally, in four of 25 sampled *Lm* localities. In these populations most individuals were introgressed and the average admixture of *Lv* genes was 8.5%. Second, in the introgressed *Lm* populations, admixture was stronger than in seven syntopic localities, where it was barely detectable. Thus current syntopy, even if it leads to occasional hybridization, as shown by a single putative F1 hybrid, does not necessarily cause introgression. This is somewhat surprising because a study of a *Lm*/*Lv* hybrid zone at microscale detected strong assortative mating but also found that syntopy was almost universally accompanied by some admixture [44]. As the four admixed *Lm* populations testify, nuclear introgression of *Lv* alleles into *Lm* populations extends beyond syntopy, but does not permeate into the core of the *Lm* range.

Local differences in the extent of introgression may be explained by several mechanisms. The introgressed populations may be simply located at the tails of local hybrid zones, and would thus be sampled entirely by chance. However other potential explanations deserve consideration. Local ecological conditions may either favor introgression or delay removal of introgressed alleles by selection [87]. Differences in abundance of species in a breeding locality may force the rarer species to hybridize due to scarcity of conspecific mates, but we have not observed this effect in syntopic populations. If species are genetically structured, as in our case, introgression may be easier between some genetic groups if their genomes harbor fewer incompatible alleles and thus intrinsic selection against hybrids is weaker or ecological/sexual adaptations are similar [24,25]. *Lv* is strongly differentiated genetically [41,88,89] and various *Lv* groups come into contact with *Lm* populations in the Carpathian Basin, and outside the Carpathian belt. If introgression is neutral, the observed pattern may result from expansion-related phenomena [4]. Under the scenario modeled by Currat et al. [90], when one species invades the range of another, neutral introgression occurs almost exclusively from the resident to the invading species. Thus, local expansion of *Lm* would bring *Lv* genes onto its genetic background. A comparison of the two isolated *Lm* populations may be instructive in this respect. Population 1 at the western margin of the species range, probably the result of postglacial or more recent expansion, has recently introgressed *Lv* mtDNA and shows clear evidence of nuclear introgression. Another isolated population (6), close to the postulated refugial area of the northern *Lm* group and possibly surviving in situ for a long time, shows no trace of nuclear introgression. Scenarios related to the Currat et al. [90] model were favored as the explanation of mtDNA introgression and replacement in *Lm* by Zieliński et al. [29].

In addition to laboratory experiments which are difficult to perform in this system due to logistic reasons, two other kinds of

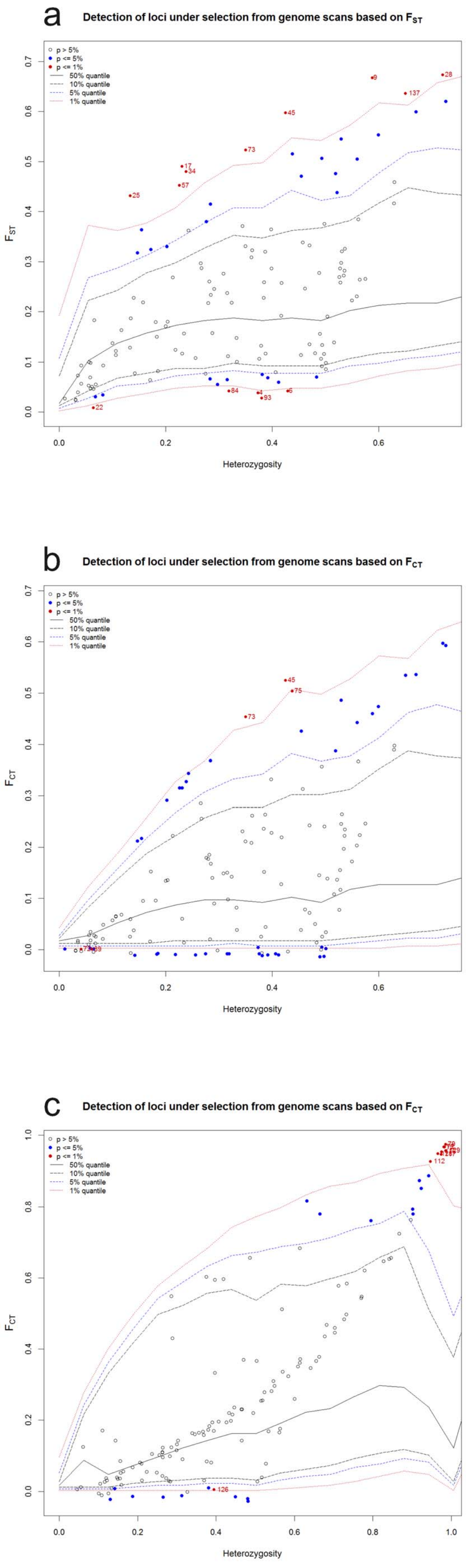

Figure 7. Detection of outlier loci from genome scans. (a) F_{ST} outliers in *L. montandoni*, (b) F_{CT} outliers in *L. montandoni*, (c) F_{CT} outliers in interspecific analysis – markers at the extremes of interspecific differentiation.
doi:10.1371/journal.pone.0097431.g007

analyses would be informative with respect to the causes of the apparent differentiation in the extent of introgression. Examination of several transects through hybrid zones in the context of local environmental conditions and relative species abundance could be informative as demonstrated in multiple systems [87,91–93]. Another important way forward would be to use multilocus sequence data [94] to construct and test multipopulation models of gene flow between *Lm* and *Lv*. Models distinguishing two groups within *Lv*, inside and outside of the Carpathian basin, as well as two groups within *Lm* can be evaluated and hypotheses regarding the timing and extent of gene flow may be tested within an Approximate Bayesian Computations framework [95,96]. This approach would provide a longer-scale perspective on gene flow between species and its spatial and temporal variation.

Genomic heterogeneity of gene flow within and between species

Outlier loci were detected both within *Lm* and between *Lm* and *Lv*. Such candidate loci may signal various forms of selection acting on the markers themselves or at linked sites [24,38]. Alternatively their apparent outlier status may result from violation of the model assumptions, to which the available methods are very sensitive [13]. We do not attempt a formal functional analysis of the identified outliers but rather emphasize that the outliers detected in the *Lm-Lv* comparison indicate heterogeneity of interspecific gene flow in nuclear protein coding genes. Dramatic discordance in the propensity for interspecific gene flow occurs between the mitochondrial and nuclear genome ([29]; this study). Within the nuclear genome the genes of MHC class II introgress easily between the two species [45]. The present study suggests that heterogeneity of gene flow is widespread in the nuclear genome. Some genomic regions, typically linked to genes involved in intergenomic incompatibilities or underlying species-specific adaptations, i.e. genes which may cause reduced hybrid fitness, acquire reproductive isolation earlier than other regions [38,97,98]. The size of such regions and mechanisms responsible for maintenance of genomic differentiation have been a subject of ongoing controversy and intense recent research [85,98–101]. It is expected that the shape of the heterogeneity in gene flow will evolve over time and a comparison of the extent of heterogeneity at various stages of divergence is of great interest for the understanding of the buildup of genomic divergence as differentiation progresses [40,86]. Transcriptome data, such as those used here for the development of SNP markers, are being applied to study genomic heterogeneity of gene flow in the *Lm*/*Lv* system (Stuglik et al. in prep.).

Conclusions

Using a panel of transcriptome-derived SNP markers, our study has demonstrated that isolation in glacial refugia and limited dispersal have been the main factors determining the genetic structure of *Lm*. Two substantially differentiated groups of *Lm* populations likely originated from separate refugia, both located in the Eastern Carpathians. The colonization of the present range in north-western and south-western directions was accompanied by a modest loss of variation. Local introgression of *Lv* alleles into several *Lm* populations was detected. Introgression was higher in

Lm populations classified as admixed than in syntopic populations. We discuss the possible causes of this discrepancy and propose further tests to distinguish between alternatives. Several outliers were identified in tests of interspecific differentiation, suggesting genomic heterogeneity of gene flow between species. The shape of genomic heterogeneity at various stages of species divergence is of major interest for the understanding of the buildup of differentiation across the genome and *Lm*/*Lv* is a promising study system in this respect.

Supporting Information

Figure S1 Expected heterozygosity. (a) interpolated geographic gradients in *L. montandoni* (*Lm*), (b) means for all populations: triangles – *Lm*, diamonds – syntopic, circles – *Lv*.
(TIF)

Figure S2 Histograms showing the distribution of pairwise F_{ST} between populations within the northern and southern *L. montandoni* groups and between groups.
(TIF)

Figure S3 Identification of the number of groups (*K*) in Structure analysis for *L. montandoni*. (a) Evanno et al. (2005) method; (b) means and standard deviations (SD) of the ln-likelihood of the probability of data for various values of *K*.
(TIF)

Figure S4 Interpolated geographic gradients of allelic richness in *L. montandoni*.
(TIF)

Figure S5 Isolation by distance in *L. montandoni*. Relationships between pairwise F_{ST} and log-geographic distances are presented for all populations and populations within northern and southern groups separately.
(TIF)

Figure S6 Identification of the number of groups (*K*) in Structure analysis for *L. montandoni* and *L. vulgaris*. (a) Evanno et al. (2005) method; (b) means and standard deviations (SD) of the ln-likelihood of the probability of data for various values of *K*.
(TIF)

Table S1 Characteristics of the single nucleotide polymorphisms (SNP) used in the present study.
(XLSX)

Table S2 Results of the tests of the Hardy-Weinberg proportions for all loci in all populations. Uncorrected *P* values are given; "-" indicates that test was not performed due to insufficient polymorphism.
(XLSX)

Table S3 Pairwise F_{ST} values. Non-significant ($P<0.05$) values marked in red.
(XLSX)

Acknowledgments

We are grateful to Maciek Bonk, Marta Chloupek, Severus Covaciu-Marcov, Dan Cogălniceanu, Magda Herdegen, Krystyna Nadachowska-Brzyska, Zofia Prokop, and Jacek Radwan who helped in sample collection. Many thanks to prof. Joanna Wesoły and dr Małgorzata Rydzanicz for the GoldenGate Genotyping (Illumina) which was performed in Genome Analysis Laboratory (Laboratory of High Throughput Technologies, IBMiB, Faculty of Biology UAM, Poznan). We are grateful to Maciej Pabijan for proofreading.

Author Contributions

Conceived and designed the experiments: WB PZ. Performed the experiments: PZ KD ML. Analyzed the data: PZ. Contributed reagents/materials/analysis tools: WB PZ KD MTS. Wrote the paper: WB PZ.

References

1. Slatkin M (1987) Gene flow and the geographic structure of natural populations. Science 236: 787–792.
2. Avise JC (2000) Phylogeography. The history and formation of species. Cambridge-London: Cambridge University Press.
3. Manel S, Holderegger R (2013) Ten years of landscape genetics. Trends in Ecology and Evolution 28: 614–621.
4. Excoffier L, Foll M, Petit RJ (2009) Genetic consequences of range expansions. Annual Review of Ecology, Evolution, and Systematics 40: 481–501.
5. Charlesworth B, Charlesworth D, Barton NH (2003) The Effects of Genetic and Geographic Structure on Neutral Variation. Annual Review of Ecology, Evolution, and Systematics 34: 99–125.
6. Bohonak AJ (1999) Dispersal, gene flow, and population structure. Quarterly Review of Biology 74: 21–45.
7. Hewitt GM (2011) Quaternary phylogeography: The roots of hybrid zones. Genetica 139: 617–638.
8. Foll M, Gaggiotti O (2006) Identifying the environmental factors that determine the genetic structure of populations. Genetics 174: 875–891.
9. Rosenberg NA, Pritchard JK, Weber JL, Cann HM, Kidd KK, et al. (2002) Genetic structure of human populations. Science 298: 2381–2385.
10. Frankham R, Ballou JD, Briscoe DA (2010) Introduction to Conservation Genetics: Cambridge Univ. Press.
11. Beaumont MA (2005) Adaptation and speciation: what can F-st tell us? Trends in Ecology & Evolution 20: 435–440.
12. Storz JF (2005) Using genome scans of DNA polymorphism to infer adaptive population divergence. Molecular Ecology 14: 671–688.
13. Narum SR, Hess JE (2011) Comparison of F ST outlier tests for SNP loci under selection. Molecular Ecology Resources 11: 184–194.
14. Frean M, Rainey PB, Traulsen A (2013) The effect of population structure on the rate of evolution. Proceedings of the Royal Society B: Biological Sciences 280.
15. Wakeley J (2001) The coalescent in an island model of population subdivision with variation among demes. Theoretical Population Biology 59: 133–144.
16. Ingvarsson PK (2004) Population subdivision and the Hudson-Kreitman-Aguade test: Testing for deviations from the neutral model in organelle genomes. Genetical Research 83: 31–39.
17. Nielsen R (2005) Molecular signatures of natural selection. Annual Review of Genetics 39: 197–218.
18. Städler T, Haubold B, Merino C, Stephan W, Pfaffelhuber P (2009) The impact of sampling schemes on the site frequency spectrum in nonequilibrium subdivided populations. Genetics 182: 205–216.
19. Hickerson MJ, Carstens BC, Cavender-Bares J, Crandall KA, Graham CH, et al. (2010) Phylogeography's past, present, and future: 10 years after Avise, 2000. Molecular Phylogenetics and Evolution 54: 291–301.
20. Hutchison DW, Templeton AR (1999) Correlation of pairwise genetic and geographic distance measures: Inferring the relative influences of gene flow and drift on the distribution of genetic variability. Evolution 53: 1898–1914.
21. Slatkin M (1993) Isolation by distance in equilibrium and non-equilibrium populations. Evolution 47: 264–279.
22. Landguth EL, Cushman SA, Schwartz MK, McKelvey KS, Murphy M, et al. (2010) Quantifying the lag time to detect barriers in landscape genetics. Molecular Ecology 19: 4179–4191.
23. Stewart JR (2009) The evolutionary consequence of the individualistic response to climate change. Journal of Evolutionary Biology 22: 2363–2375.
24. Bierne N, Welch J, Loire E, Bonhomme F, David P (2011) The coupling hypothesis: Why genome scans may fail to map local adaptation genes. Molecular Ecology 20: 2044–2072.
25. Corbett-Detig RB, Zhou J, Clark AG, Hartl DL, Ayroles JF (2013) Genetic incompatibilities are widespread within species. Nature 504: 135–137.
26. Kisel Y, Timothy G. Barraclough TG (2010) Speciation has a spatial scale that depends on levels of gene flow. American Naturalist 175: 316–334.
27. Smith MA, Green DM (2005) Dispersal and the metapopulation paradigm in amphibian ecology and conservation: Are all amphibian populations metapopulations? Ecography 28: 110–128.
28. Vences M, Wake DB (2007) Speciation, species boundaries and phylogeography of amphibians. In: Heatwole H, Tyler M, editors. Amphibian Biology, Vol

6, Systematics. Chipping Norton, Australia: Surrey Beatty & Sons.pp. 2613–2669.
29. Zieliński P, Nadachowska-Brzyska K, Wielstra B, Szkotak R, Covaciu-Marcov SD, et al. (2013) No evidence for nuclear introgression despite complete mtDNA replacement in the Carpathian newt (Lissotriton montandoni). Molecular Ecology 22: 1884–1903.
30. Brumfield RT, Beerli P, Nickerson DA, Edwards SV (2003) The utility of single nucleotide polymorphisms in inferences of population history. Trends in Ecology and Evolution 18: 249–256.
31. Helyar SJ, Hemmer-Hansen J, Bekkevold D, Taylor MI, Ogden R, et al. (2011) Application of SNPs for population genetics of nonmodel organisms: New opportunities and challenges. Molecular Ecology Resources 11: 123–136.
32. Fan JB, Chee MS, Gunderson KL (2006) Highly parallel genomic assays. Nature Reviews Genetics 7: 632–644.
33. Davey JW, Hohenlohe PA, Etter PD, Boone JQ, Catchen JM, et al. (2011) Genome-wide genetic marker discovery and genotyping using next-generation sequencing. Nature Reviews Genetics 12: 499–510.
34. Clark AG, Hubisz MJ, Bustamante CD, Williamson SH, Nielsen R (2005) Ascertainment bias in studies of human genome-wide polymorphism. Genome Research 15: 1496–1502.
35. Paschou P, Ziv E, Burchard EG, Choudhry S, Rodriguez-Cintron W, et al. (2007) PCA-correlated SNPs for structure identification in worldwide human populations. PLoS Genetics 3: 1672–1686.
36. Haasl RJ, Payseur BA (2011) Multi-locus inference of population structure: A comparison between single nucleotide polymorphisms and microsatellites. Heredity 106: 158–171.
37. Rosenblum EB, Novembre J (2007) Ascertainment bias in spatially structured populations: A case study in the Eastern Fence Lizard. Journal of Heredity 98: 331–336.
38. Nosil P, Funk DJ, Ortiz-Barrientos D (2009) Divergent selection and heterogeneous genomic divergence. Molecular Ecology 18: 375–402.
39. Sousa V, Hey J (2013) Understanding the origin of species with genome-scale data: Modelling gene flow. Nature Reviews Genetics 14: 404–414.
40. Roux C, Tsagkogeorga G, Bierne N, Galtier N (2013) Crossing the species barrier: Genomic hotspots of introgression between two highly divergent ciona intestinalis species. Molecular Biology and Evolution 30: 1574–1587.
41. Babik W, Branicki W, Crnobrnja-Isailović J, Cogălniceanu D, Sas I, et al. (2005) Phylogeography of two European newt species - Discordance between mtDNA and morphology. Molecular Ecology 14: 2475–2491.
42. Provan J, Bennett KD (2008) Phylogeographic insights into cryptic glacial refugia. Trends in Ecology and Evolution 23: 564–571.
43. Tzedakis PC, Emerson BC, Hewitt GM (2013) Cryptic or mystic? Glacial tree refugia in northern Europe. Trends in Ecology & Evolution 28: 696–704.
44. Babik W, Szymura JM, Rafiński J (2003) Nuclear markers, mitochondrial DNA and male secondary sexual traits variation in a newt hybrid zone (Triturus vulgaris x T. montandoni). Molecular Ecology 12: 1913–1930.
45. Nadachowska-Brzyska K, Zieliński P, Radwan J, Babik W (2012) Interspecific hybridization increases MHC class II diversity in two sister species of newts. Molecular Ecology 21: 887–906.
46. Landguth EL, Fedy BC, Oyler-Mccance SJ, Garey AL, Emel SL, et al. (2012) Effects of sample size, number of markers, and allelic richness on the detection of spatial genetic pattern. Molecular Ecology Resources 12: 276–284.
47. Grabherr MG, Haas BJ, Yassour M, Levin JZ, Thompson DA, et al. (2011) Full-length transcriptome assembly from RNA-Seq data without a reference genome. Nature Biotechnology 29: 644–652.
48. Stuglik MT, Babik W, Prokop Z, Radwan J (2014) Alternative reproductive tactics and sex-biased gene expression: the study of the bulb mite transcriptome. Ecology and Evolution. pp. 623–632.
49. Langmead B, Salzberg SL (2012) Fast gapped-read alignment with Bowtie 2. Nature Methods 9: 357–359.
50. Li H, Handsaker B, Wysoker A, Fennell T, Ruan J, et al. (2009) The Sequence Alignment/Map format and SAMtools. Bioinformatics 25: 2078–2079.
51. Edgar RC, Haas BJ, Clemente JC, Quince C, Knight R (2011) UCHIME improves sensitivity and speed of chimera detection. Bioinformatics 27: 2194–2200.
52. Rousset F (2008) GENEPOP'007: A complete re-implementation of the GENEPOP software for Windows and Linux. Molecular Ecology Resources 8: 103–106.
53. Storey JD (2002) A direct approach to false discovery rates. Journal of the Royal Statistical Society Series B: Statistical Methodology 64: 479–498.
54. Storey JD, Tibshirani R (2003) Statistical significance for genomewide studies. Proceedings of the National Academy of Sciences of the United States of America 100: 9440–9445.
55. Jombart T (2008) Adegenet: A R package for the multivariate analysis of genetic markers. Bioinformatics 24: 1403–1405.
56. Goudet J (1995) FSTAT (Version 1.2): A computer program to calculate F-statistics. Journal of Heredity 86: 485–486.
57. Excoffier L, Lischer HEL (2010) Arlequin suite ver 3.5: A new series of programs to perform population genetics analyses under Linux and Windows. Molecular Ecology Resources 10: 564–567.
58. Jensen JL, Bohonak AJ, Kelley ST (2005) Isolation by distance, web service. BMC Genetics 6: 13.
59. Takezaki N, Nei M, Tamura K (2010) POPTREE2: Software for constructing population trees from allele frequency data and computing other population statistics with windows interface. Molecular Biology and Evolution 27: 747–752.
60. Latter BD (1972) Selection in finite populations with multiple alleles. 3. Genetic divergence with centripetal selection and mutation. Genetics 70: 475–490.
61. Pritchard JK, Stephens M, Donnelly P (2000) Inference of population structure using multilocus genotype data. Genetics 155: 945–959.
62. Falush D, Stephens M, Pritchard JK (2007) Inference of population structure using multilocus genotype data: Dominant markers and null alleles. Molecular Ecology Notes 7: 574–578.
63. Hubisz MJ, Falush D, Stephens M, Pritchard JK (2009) Inferring weak population structure with the assistance of sample group information. Molecular Ecology Resources 9: 1322–1332.
64. Evanno G, Regnaut S, Goudet J (2005) Detecting the number of clusters of individuals using the software STRUCTURE: A simulation study. Molecular Ecology 14: 2611–2620.
65. Earl DA, vonHoldt BM (2012) STRUCTURE HARVESTER: a website and program for visualizing STRUCTURE output and implementing the Evanno method. Conservation Genetics Resources 4: 359–361.
66. Slatkin M, Voelm L (1991) F(ST) in a hierarchical island model. Genetics 127: 627–629.
67. Excoffier L, Hofer T, Foll M (2009) Detecting loci under selection in a hierarchically structured population. Heredity 103: 285–298.
68. François O, Durand E (2010) Spatially explicit Bayesian clustering models in population genetics. Molecular Ecology Resources 10: 773–784.
69. Frantz AC, Cellina S, Krier A, Schley L, Burke T (2009) Using spatial Bayesian methods to determine the genetic structure of a continuously distributed population: Clusters or isolation by distance? Journal of Applied Ecology 46: 493–505.
70. Li JZ, Absher DM, Tang H, Southwick AM, Casto AM, et al. (2008) Worldwide human relationships inferred from genome-wide patterns of variation. Science 319: 1100–1104.
71. Sommer RS, Nadachowski A (2006) Glacial refugia of mammals in Europe: Evidence from fossil records. Mammal Review 36: 251–265.
72. Ronikier M (2011) Biogeography of high-mountain plants in the Carpathians: An emerging phylogeographical perspective. Taxon 60: 373–389.
73. Hewitt GM (1999) Post-glacial re-colonization of European biota. Biological Journal of the Linnean Society 68: 87–112.
74. Bayger JA (1937) Klucz do oznaczania płazów i gadów. Kraków: Koło Przyrodników Studentów Uniwersytetu Jagiellońskiego.
75. Litvinchuk SN, Borkin LJ, Rosanov JM (2003) On distribution of and hybridization between the newts *Triturus vulgaris* and *T. montandoni* in western Ukraine. Alytes 20. pp. 161–168.
76. Beebee TJC (1996) Ecology and conservation of amphibians. London: Chapman & Hall.
77. Cabe PR, Page RB, Hanlon TJ, Aldrich ME, Connors L, et al. (2007) Fine-scale population differentiation and gene flow in a terrestrial salamander (Plethodon cinereus) living in continuous habitat. Heredity 98: 53–60.
78. Helfer V, Broquet T, Fumagalli L (2012) Sex-specific estimates of dispersal show female philopatry and male dispersal in a promiscuous amphibian, the alpine salamander (Salamandra atra). Molecular Ecology 21: 4706–4720.
79. Savage WK, Fremier AK, Shaffer HB (2010) Landscape genetics of alpine Sierra Nevada salamanders reveal extreme population subdivision in space and time. Molecular Ecology 19: 3301–3314.
80. Mullen LB, Woods HA, Schwartz MK, Sepulveda AJ, Lowe WH (2010) Scale-dependent genetic structure of the Idaho giant salamander (Dicamptodon aterrimus) in stream networks. Molecular Ecology 19: 898–909.
81. Steele CA, Baumsteiger J, Storfer A (2009) Influence of life-history variation on the genetic structure of two sympatric salamander taxa. Molecular Ecology 18: 1629–1639.
82. Unger SD, Rhodes Jr OE, Sutton TM, Williams RN (2013) Population Genetics of the Eastern Hellbender (Cryptobranchus alleganiensis alleganiensis) across Multiple Spatial Scales. PLoS ONE 8.
83. Sotiropoulos K, Eleftherakos K, Tsaparis D, Kasapidis P, Giokas S, et al. (2013) Fine scale spatial genetic structure of two syntopic newts across a network of ponds: Implications for conservation. Conservation Genetics 14: 385–400.
84. Vähä JP, Primmer CR (2006) Efficiency of model-based Bayesian methods for detecting hybrid individuals under different hybridization scenarios and with different numbers of loci. Molecular Ecology 15: 63–72.
85. Flaxman SM, Feder JL, Nosil P (2013) Genetic hitchhiking and the dynamic buildup of genomic divergence during speciation with gene flow. Evolution 67: 2577–2591.
86. Nadeau NJ, Whibley A, Jones RT, Davey JW, Dasmahapatra KK, et al. (2012) Genomic islands of divergence in hybridizing heliconius butterflies identified by large-scale targeted sequencing. Philosophical Transactions of the Royal Society B: Biological Sciences 367: 343–353.
87. Nolte AW, Gompert Z, Buerkle CA (2009) Variable patterns of introgression in two sculpin hybrid zones suggest that genomic isolation differs among populations. Molecular Ecology 18: 2615–2627.
88. Rafiński J, Cogălniceanu D, Babik W (2001) Genetic differentiation of the two subspecies of the smooth newt inhabiting Romania, Triturus vulgaris vulgaris and T. v. ampelensis (Urodela, Salamandridae) as revealed by enzyme electrophoresis. Folia Biologica 49: 239–245.

89. Nadachowska K, Babik W (2009) Divergence in the face of gene flow: The case of two newts (Amphibia: Salamandridae). Molecular Biology and Evolution 26: 829–841.
90. Currat M, Ruedi M, Petit RJ, Excoffier L (2008) The hidden side of invasions: Massive introgression by local genes. Evolution 62: 1908–1920.
91. Dowling TE, Broughton RE, Demarais BD (1997) Significant role for historical effects in the evolution of reproductive isolation: Evidence from patterns of introgression between the cyprinid fishes, Luxilus cornutus and Luxilus chrysocephalus. Evolution 51: 1574–1583.
92. Aboim MA, Mavárez J, Bernatchez L, Coelho MM (2010) Introgressive hybridization between two Iberian endemic cyprinid fish: A comparison between two independent hybrid zones. Journal of Evolutionary Biology 23: 817–828.
93. Teeter KC, Thibodeau LM, Gompert Z, Buerkle CA, Nachman MW, et al. (2010) The variable genomic architecture of isolation between hybridizing species of house mice. Evolution 64: 472–485.
94. Zieliński P, Stuglik MT, Dudek K, Konczal M, Babik W (2014) Development, validation and high-throughput analysis of sequence markers in nonmodel species. Molecular Ecology Resources 14: 352–360.
95. Beaumont MA (2010) Approximate Bayesian computation in evolution and ecology. Annual Review of Ecology, Evolution, and Systematics 41: 379–406.
96. Nadachowska-Brzyska K, Burri R, Olason PI, Kawakami T, Smeds L, et al. (2013) Demographic Divergence History of Pied Flycatcher and Collared Flycatcher Inferred from Whole-Genome Re-sequencing Data. PLoS Genetics 9.
97. Wu CI, Ting CT (2004) Genes and speciation. Nature Reviews Genetics 5: 114–122.
98. Nosil P, Feder JL (2012) Genomic divergence during speciation: Causes and consequences. Philosophical Transactions of the Royal Society B: Biological Sciences 367: 332–342.
99. Noor MAF, Bennett SM (2009) Islands of speciation or mirages in the desert Examining the role of restricted recombination in maintaining species. Heredity 103: 439–444.
100. Lawniczak MKN, Emrich SJ, Holloway AK, Regier AP, Olson M, et al. (2010) Widespread divergence between incipient Anopheles gambiae species revealed by whole genome sequences. Science 330: 512–514.
101. Via S (2012) Divergence hitchhiking and the spread of genomic isolation during ecological speciation-with-gene-flow. Philosophical Transactions of the Royal Society B: Biological Sciences 367: 451–460.
